# Synthesis of New 2,5-Di-substituted 1,3,4-Oxadiazoles Bearing 2,6-Di-*tert*-butylphenol Moieties and Evaluation of Their Antioxidant Activity

**DOI:** 10.3390/molecules19033436

**Published:** 2014-03-20

**Authors:** Raied M. Shakir, Azhar Ariffin, Mahmood Ameen Abdulla

**Affiliations:** 1 Department of Chemistry, Faculty of Science, University of Malaya, Kuala Lumpur 50603, Malaysia; E-Mail: raiedalsayab@yahoo.co.uk; 2 Department of Chemistry, Ibn Al-haitham, University of Baghdad, Baghdad 61023, Iraq; 3 Department of Molecular Medicine, Faculty of Medicine, University of Malaya, Kuala Lumpur 50603, Malaysia; E-Mail: ammeen@um.edu.my

**Keywords:** 2,6-di-*tert*-butylphenol, hindered phenol, antioxidant, 1,3,4-oxadiazole, FRAP, DPPH

## Abstract

Eleven new 2,6-di-*tert*-butyl-4-(5-aryl-1,3,4-oxadiazol-2-yl)phenols **5a**–**k** were synthesized by reacting aryl hydrazides with 3,5-di-*tert* butyl 4-hydroxybenzoic acid in the presence of phosphorus oxychloride. The resulting compounds were characterized based on their IR, ^1^H-NMR, ^13^C-NMR, and HRMS data. 2,2-Diphenyl-1-picrylhydrazide (DPPH) and ferric reducing antioxidant power (FRAP) assays were used to test the antioxidant properties of the compounds. Compounds **5f** and **5j** exhibited significant free-radical scavenging ability in both assays.

## 1. Introduction

**Figure 1:**
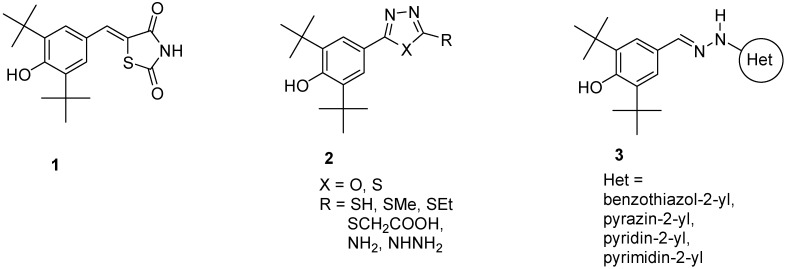
Di-*tert*-butyl-phenols with long-chain resonance as antioxidants.

Phenolic antioxidants inhibit or prevent oxidative stress in biological systems. Free radicals are one of the main causes of many pathological conditions such as those that cause several degenerative [1] and chronic diseases [2]. Furthermore, numerous heterocyclic compounds containing di-*tert*-butyl phenol exhibit various types of biological activity in addition to their antioxidant ability [3,4]. Cyclo-oxygenase and 5-lipoxygenase [5,6] exhibit anti-inflammatory [3,7] and anticancer [8,9] activities. Many synthesized compounds contain long-chain resonance and exhibit high antioxidant activity, such as **1**, **2**, and **3** [10,11,12] (Figure 1).

Antioxidants donate protons to become a stable free radicals. This stability increases with the extent of delocalisation [13] and enhances antioxidant ability [14]. A number of 1,3,4-oxadiazole derivatives have exhibited various types of biological activity [15,16,17] and antioxidant ability [18,19].

**Scheme 1:**
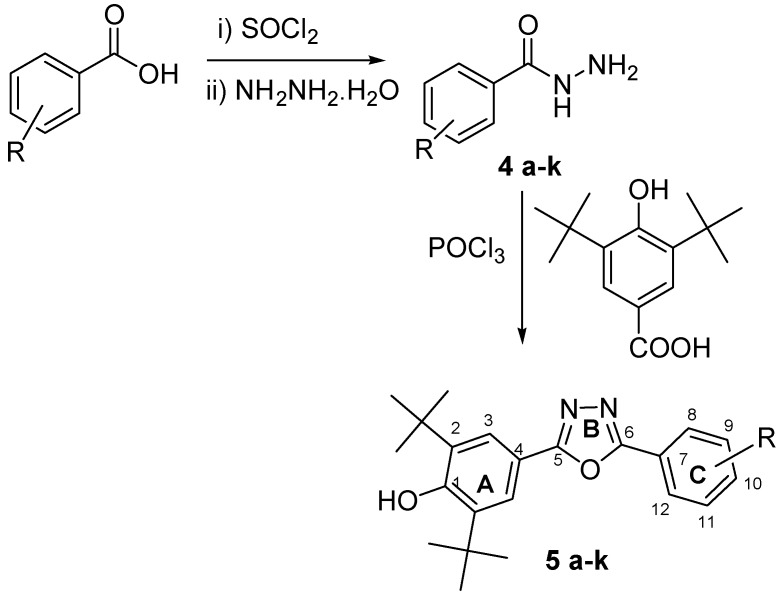
Synthesis of 2,6-di-*tert*-butyl-4-(5-aryl-1,3,4-oxadiazol-2-yl)phenols.

This paper describes the synthesis of eleven 2,6-di-*tert*-butyl-4-(5-aryl-1,3,4-oxadiazole-2-yl)phenols **5a**–**k** (Scheme 1) and evaluates their antioxidant activity. These new oxadiazoles are designed to be effective antioxidants owing to their long-chain resonance. This study also investigates the effects of different substituents on the phenyl ring C at position 6 of the 1,3,4-oxadiazole. This structure is likely to possess superior antioxidant activity compared to the 2,6-di-*tert*-butyl phenol given the enhanced stability of the free radical of the 1,3,4-oxadiazole as a result of resonance. The inductive and resonance effects could have a major role in enhancing the scavenging ability of the compound. The antioxidant ability of these compounds were measured by means of FRAP and DPPH assays.

## 2. Results and Discussion

### Chemistry

Eleven aromatic acids were converted to their corresponding aryl hydrazides **4a**–**k** by reacting the corresponding aromatic acids with thionyl chloride and then with hydrazine hydrate in dry benzene at 0 °C. The aryl hydrazides were then reacted with 3,5-di-*tert* butyl 4-hydroxybenzoic acid in the presence of POCl_3_ as dehydrating agent to obtain new 2,6-di-*tert*-butyl-4-(5-aryl-1,3,4-oxadiazol-2-yl)phenols, as demonstrated in Scheme 1. Table 1 shows the aryl groups with the corresponding yields and HREIM data.

**Table 1:** Synthesized compounds, yields, molecular formulas (MFs) and HRMS data.

No.	Compounds	Yield %	MF	HREIMS found	HREIMS calc.
**5a**	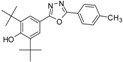	76.1	C_23_H_28_N_2_O_2_	364.2147	364.2151
**5b**	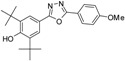	76	C_23_H_28_N_2_O_3_	380.2095	380.2100
**5c**	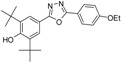	84.2	C_24_H_30_N_2_O_3_	394.2249	394.2256
**5d**	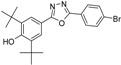	73.6	C_22_H_25_BrN_2_O_2_	428.1093	428.1099
**5e**	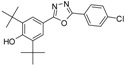	83.1	C_22_H_25_ClN_2_O_2_	384.1597	384.1605
**5f**	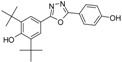	70	C_22_H_26_N_2_O_3_	366.1938	366.1943
**5g**	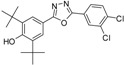	80.3	C_22_H_24_Cl_2_N_2_O_2_	418.1219	418.1215
**5h**	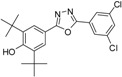	74.5	C_22_H_24_Cl_2_N_2_O_2_	418.1210	418.1215
**5i**	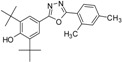	59.9	C_24_H_30_N_2_O_2_	378.2301	378.2304
**5j**	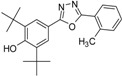	68.4	C_23_H_28_N_2_O_2_	364.2144	364.2151
**5k**	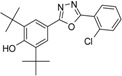	81	C_22_H_25_ClN_2_O_2_	384.1600	384.1605

**Figure 2:**
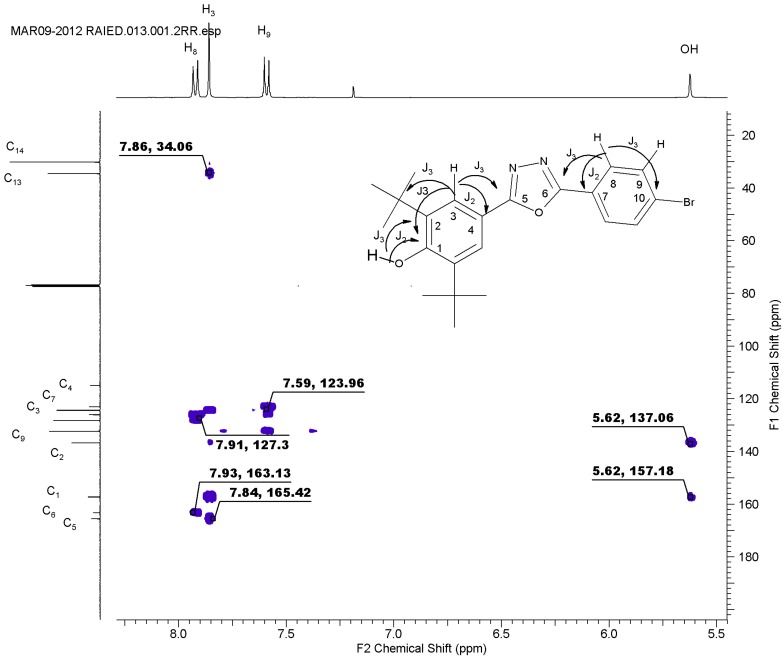
HMBC expansion region of **5d**.

The structures of the compounds were established on the basis of their spectral data. The IR exhibited all the expected peaks, with the OH of the hindered phenol showing medium to strong peaks due to nonhydrogen bonding [20] at 3658–3525, CH_aliphatic_ ones at 2963–2947, and the C=N of the oxadiazole ring at 1624 cm^−1^ to 1608 cm^−1^. The ^1^H-NMR spectra displayed the di-tert butyl group with integration equal to 18H, whose chemical shift ranged between 1.44 ppm and 1.52 ppm. The OH of the hindered phenol appeared at δ5.63 to δ5.69. All aryl protons and their substituents appeared in the expected regions. The ^13^C-NMR spectra were consistent with the IR and ^1^H-NMR spectra and our expectations. The carbons of the oxadiazole ring appeared at 161 ppm to 166 ppm, which represented 2(C=N). HMBC was employed to distinguish between C_5_ and C_6_ through the long-range coupling *J*_3_. The weak coupling *J*_2_ was also determined (Figure 2), and it exhibited the most significant correlations for the aromatic area.

H_3_ exhibited correlations with C_5_ and C_1_ and weak correlations with C_2_ and C_4_. H_8_ exhibited a correlation with C_6_ and C_10_ owing to *J*_3_, and with C_7_ and C_9_ owing to *J*_2_. This spectrum confirmed that the C_5_ of the oxadiazole appeared at a lower field than C_6_. The HREIM values for all synthesized compounds were consistent with the calculated mass and the molecular formula. For more details, see the Experimental section.

**Scheme 2:**
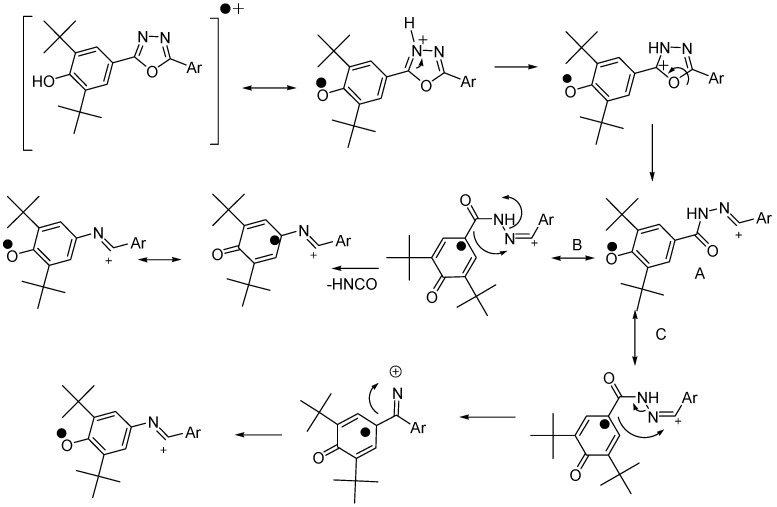
Proposed pathways of HNCO loss.

The EIMs show the molecular ion M^•+^ for all compounds and the base peak (100%) were either the same value of the molecular ion or the molecular ion minus methyl radical [M^•+^−^•^CH_3_]. The fragmentations in EIMs confirm the proposed structures and the HREIMs confirmed the accurate mass and the molecular formula. The interesting fragmentation observed in the mass spectrum was the loss of isocyanic acid (HNCO). This fragmentation which strated from [M^•+^+H] was reported in literature [21,22,23]. The oxadiazoles loose HNCO but not phenol. However, in our case, losing HNCO started from molecular ion (M^•+^) which was subsequently protonated. The loss of HNCO can be explained through the rearrangement of the molecular ion and migration. Scheme 2 describes the proposed mechanism of the elimination of isocyanic acid.

Two pathways were suggested for the radical migration, path (B) and (C). Both pathways, (B) and (C) have intermediate (A) as their starting point which is formed by intra fragmentation of the molecular ion and migration of 2,6-di-*tert*-butylphenol. Path B is similar to the mass fragmentation pathway proposed by Frański *et al*. [22]. Table 2 summarizes the value of the molecular ion, the base peak and value after losing HNCO.

**Table 2:** Molecular ion found, calculated, base peak and the *m/z* after losing HNCO.

5	M^•+^ Found	M^•+^ calculated	*m/z* of base peak 100%	M^•+^-HNCO
**5a**	364.2	364.21	349.2	321.1
**5b**	380.2	380.21	380.2	337.1
**5c**	394.3	394.22	394.3	351.1
**5d**	428.2	428.10	413.1	385.1
**5e**	384.2	384.16	369.1	341.1
**5f**	366.2	366.19	366.2	323.1
**5g**	418.2	418.12	403.1	375.1
**5h**	418.2	418.12	403.1	375.0
**5i**	378.3	378.23	378.3	335.1
**5j**	364.3	364.21	349.2	321.1
**5k**	384.2	384.16	369.2	341.1

## 3. Antioxidant Assays

### 3.1. FRAP Assay

The FRAP assay was performed according to the Benzie and Strain [24] method. The FRAP reagent was prepared by combining 300 mM acetate buffer and 10 mM 2,4,6-tripyridyl-*s*-triazine (TPTZ) solution in 40 mM HCl and 20 mM FeCl_3_·6H_2_O, in a ratio of 10:1:1. The FRAP reagent was incubated at 37 °C prior to use. Ten microliters of the sample was reconstituted in the carrier (solvent or ultrapure water) and mixed with 300 μL of FRAP reagent. The mixture was incubated at 37 °C for 4 min in a microplate reader. The absorbance of the complex was 593 nm. The FRAP value can be calculated using the following equation [25]:

FRAP = [(0–4 min ∆A593 nm of test sample)/(0–4 min ∆A593 nm of standard)] × [standard] (µM) × Y × 1000

where Y is absorbance of the spectrophotometer.

### 3.2. DPPH Assay

The assay was performed as reported by Gerhauser *et al*. [26]. Five microliters of the sample (dissolved in ethanol) was added into 195 μL of 100 μM DPPH reagent in ethanol (96%) and mixed in a 96-well plate. The intensity of the color was measured for 3 h at an interval of 20 min at 515 nm. Ascorbic acid and BHT were used as reference.

### 3.3. Antioxidant Activity

Differences occurred between the structures of the synthesized compound in ring C owing to different substituent and positions, whereas rings A and B were the same. Various antioxidant abilities were exhibited in both assays based on the type of substituent and their position, which have important roles in enhancing or negating antioxidant ability. The inductive effects of the electron-donating group +I and electron-withdrawing group (EWG) −I, the mesomeric effect (electron-releasing group +M or electron-withdrawing group −M), and the resonance effect directly affected antioxidant ability. Compound **5f** exhibited higher antioxidant capacity, with a FRAP value of 2207.25 (see Table 3). This result is consistent with the concept that the hydroxyl group enhances antioxidant ability [27,28,29,30]. The +M of the hydroxyl at the *para* position is more important than the −I. Compound **5j** exhibited excellent antioxidant ability (1538.9).

**Figure 3:**
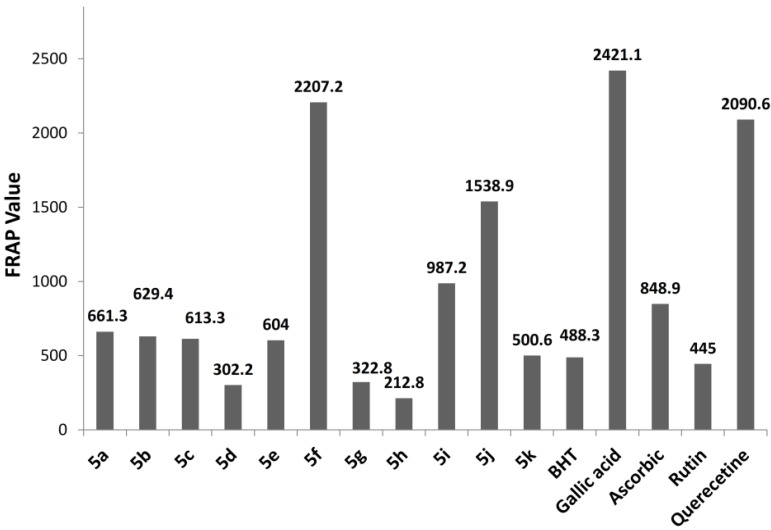
FRAP assay for the synthesized 2,6-di-*tert*-butyl-4-(5-aryl-1,3,4-oxadiazol-2-yl)phenols.

The FRAP value and the substituent followed the following sequence: 4-OH > 2-Me > 2,4-di-Me > 4-Me > 4-OMe ≈ 4-OEt > 4-Cl> 2-Cl > 4-Br ≈ 3,4-di Cl ≈ 3,5-di Cl. This sequence demonstrates that the electron-releasing group, which exerts mesomeric and inductive effects, enhances antioxidant ability, whereas the inductive-withdrawing group decreases antioxidant ability. The results show that the position of substituent also affects antioxidant ability, as illustrated in Figure 3.

**Table 3:** Antioxidant activity of the synthesized oxadiazoles.

Compound	FRAP ^a^	DPPH Inhibition % ±SD	IC_50_ ± SEM ^b^ (100 µg/mL)
**5a**	648.3	76.02 ± 0.059	41.76 ± 0.042
**5b**	629.4	62.03 ± 0.327	50.69 ± 0.181
**5c**	613.3	56.04 ± 0.187	54.60 ± 0.469
**5d**	302.2	30.85 ± 0.166	>100
**5e**	640.0	50.44 ± 0.045	99.2 ± 0.032
**5f**	2207.2	89.05 ± 0.024	15.79 ± 0.017
**5g**	322.8	30.35 ± 0.038	>100
**5h**	212.8	29.26 ± 0.041	>100
**5i**	987.2	79.22 ± 0.037	41.27 ± 0.027
**5j**	1538.9	87.21 ± 0.084	15.9 ± 0.054
**5k**	500.6	42.14 ± 0.078	>100
**BHT**	488.3	66.03 ± 0.051	79.84 ± 0.036
**Gallic acid**	2421.1	-	-
**Ascorbic acid**	848.9	90.65 ± 0.122	22.71 ± 0.086
**Rutin**	445.0	-	-
**Quercetin**	2090.6	-	-
**Trolox**	779.4	-	-

^a^ Standard deviation (SD) value in FRAP was between 0.01–0.16; ^b^ SED standard mean error and IC_50_: 50% effective concentration.

The methyl group at position 2 enhances the antioxidant ability more than that at position 4, and exhibits higher antioxidant ability than 4-methoxy. However, the differences between 4-methoxy and 4-ethoxy were too small. The analogue with EWG in positions 2, 3, and 4, *i.e.*, 4-Br, 4-Cl, 3,4-diCl, 3,5-diCl reduced or negated antioxidant activity. The results may be explained by the fact that previously described analogues with EWG’s, increase the bond dissociation energy. Another possibility is that they exhibit decreased antioxidant ability [31,32]. The DPPH results (Table 3) were compatible with, and possessed the same sequence, as the FRAP assay. However, all values for DPPH are on the whole lower than the values for the FRAP assay in comparison to ascorbic acid. For instance, in FRAP assay, **5f** showed about twice the antioxidant ability of ascorbic acid, whereas in the DPPH assay, **5f** exhibited similar antioxidant ability to ascorbic acid. This difference could be attributed to the different mechanisms for FRAP and DPPH. FRAP involves the single electron transfer mechanism, whereas DPPH assay depends on the H-atom transfer mechanism [33]. The steric hindrance between the synthesized compound and DPPH may account for the difference [34].

**Figure 4:**
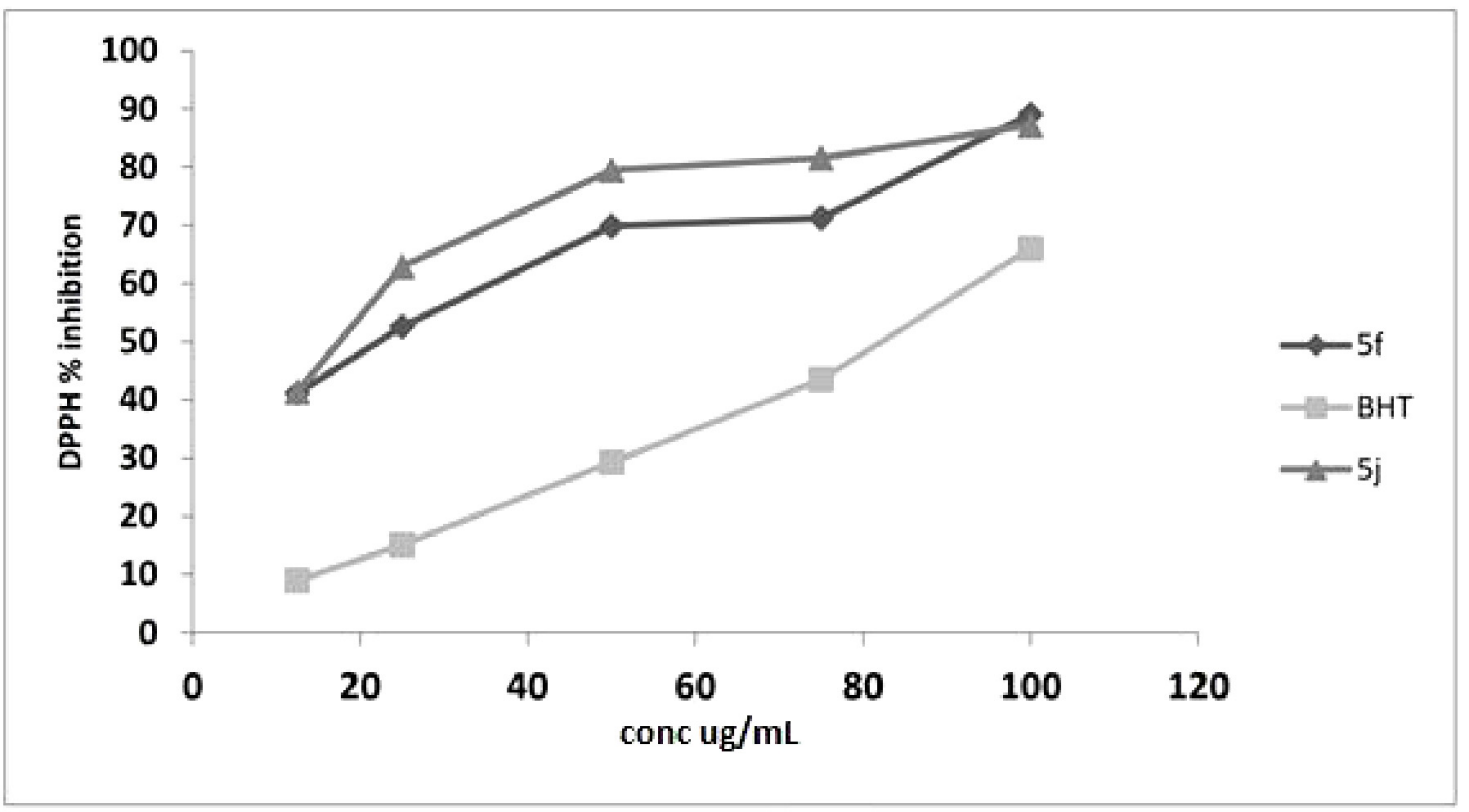
DPPH inhibitions of **5f** and **5j** at different concentration.

Compounds **5f** and **5j** exhibited lower IC_50_ values of 15.79 and 15.9 µg/mL, respectively, compared to ascorbic acid. The IC_50_ of *para* substituted analogues with methyl, methoxy, and ethoxy were lower than that of BHT. By contrast, for electron withdrawing group at *para*, *meta*, and *ortho*, the analogues showed reduced antioxidant activity. All compounds were screened in terms of their free radical scavenging properties using five concentrations: 12.5, 25, 50, 75, and 100 µg/mL. Both **5f** and **5j** exhibited significant antioxidant capability at low concentrations, as depicted in Figure 4.

## 4. Experimental

### General Information

The chemicals used for the synthesis were supplied by Sigma-Aldrich (Petaling Jaya, Selangor, Malaysia), Fisher (Shah Alam, Selangor, Malaysia), and Merck (Petaling Jaya, Selangor, Malaysia). The melting point was determined by open capillary tube method using an MEL-TEMP II apparatus and was uncorrected. The purity of the compounds was checked through thin layer chromatography (silica gel TLC) using Merck plates. The plates were visualized by mean of iodine vapors and UV light. The IR spectra were obtained using a PerkinElmer 400 Fourier transform infrared spectrometer. All NMR spectra were recorded on either a JEOL-ECA 400 MHz or JEOL-Lambda 400 MHz spectrometer. CDCl_3_ and DMSO-*d*_6_ were used as solvents with TMS as the internal standard. Mass spectra were recorded using a TSQ7000 for HREI/MS (NUS Singapore). For UV spectroscopy, a Power Wave X340 (BIO-TEK Instruments, Inc., Winooski, VT, USA) was used to record the FRAP and DPPH assays.

#### General Synthesis of 2,6-Di-*tert*-butyl-4-(5-aryl-1,3,4-oxadiazol-2-yl)phenols

To a mixture of (0.31 g, 1.24 mmol) of 3,5-di-*tert*-butyl-4-hydroxybenzoic acid and 1.24 mmole aryl acid hydrazide in a 50 mL round bottom flask, 5 mL of phosphorus oxychloride was added in a few portions at room temperature. The mixture was refluxed for 3 h with stirring on water bath 80–90 °C. After cooling, the mixture poured onto 100 mL crushed ice and stirred for 15 min. Sodium bicarbonate was added in a few portions until the pH was around to 7–8. The precipitate was filtered, washed with water and dried then purified either by column chromatography or by crystallization from suitable solvent.

*2,6-Di-tert-butyl-4-[5-(4-methylphenyl)-1,3,4-oxadiazol-2-yl]phenol* (**5a**). The product was recrystallized from chloroform-ethanol (1-1) to obtain white crystals. Yield 0.343 g (76.0%), m.p. 196–197 °C, IR (KBr, ν_max_/cm^−1^): 3658 (OH), 3011 (CH_aromatic_), 2962–2947 (CH_aliphatic_), 1610 (C=N), 1585, 1498 (C=C), 1219 (C-O), ^1^H-NMR (CDCl_3_, 400 MHz, ppm): 1.51 (s, 18H, H_14_, 2 × *t*-Bu), 2.42 (s, 3H, *p*-CH_3_-ph), 5.67 (s, 1H, OH), 7.31 (d, 2H, *J* = 8.28 Hz, H_9_, H_11_), 7.94 (s, 2H, H_3_), 8.01 (d, 2H, *J* = 8.28 Hz, H_8_, H_12_), ^13^C-NMR (CDCl_3_, 100 MHz, ppm): 21.73 (*p*-CH_3_Ph), 30.25 (6C, C_14_, 2 × C(CH_3_)_3_), 34.56 (2C, C_13_, 2 × C(CH_3_)_3_), 115.43 (C_4_), 121.52 (C_7_), 124.35 (C_3_), 126.87 (2C, C_8_ & C_12_), 129.77 (2C, C_9_ & C_11_), 136.81 (C_2_), 141.99 (C_10_), 157.12 (C_1_), 164.22 & 165.21 (C_5_ & C_6_). HREIMs, *m/z* = 364.2147 [M^•+^] (calc. for C_23_H_28_O_2_N_2_, 364.2151).

*2,6-Di-tert-butyl-4-[5-(4-methox yphenyl)-1,3,4-oxadiazol-2-yl]phenol* (**5b**). The solid product was recrystallized from ethyl acetate to yield white crystals. Yield 0.346 g (73%), m.p. 179–181 °C, IR (KBr, ν_max_/cm^−1^): 3625 (OH_phenol_), 3006 (CH_aromatic_), 2955 (CH_aliphatic_), 1611 (C=N), 1585, 1495 (C=C), 1219 (C-O), 1020 (O-CH_3_), ^1^H-NMR (CDCl_3_, 400 MHz, ppm): 1.51 (s, 18H, H_14_, 2 × *t*-Bu), 3.89 (s, 3H, OCH_3_), 5.63 (s, 1H, OH), 7.03 (d, 2H, *J* = 9.04 Hz, H_9_, H_11_), 7.92 (s, 2H, H_3_), 8.06 (d, 2H, *J* = 8.56 Hz, H_8_, H_12_), ^13^C-NMR (CDCl_3_, 100 MHz, ppm): 30.13 (6C, C_14_, 2 × C(CH_3_)_3_), 34.45 (2C, C_13_, 2 × C(CH_3_)_3_), 55.44 (OCH_3_), 114.40 (2C, C_8_ & C_12_), 115.37 (C_4_), 116.72 (C_7_), 124.19 (C_3_), 128.58 (2C, C_9_ & C_11_), 136.68 (C_2_), 156.95 (C_1_), 162.11 (C_10_), 163.89 & 164.75 (C_6_ & C_5_). HREIMs, *m/z* = 380.2095 [M^•+^] (calc. for C_23_H_28_O_3_N_2_, 380.2100).

*2,6-Di-tert-butyl-4-[5-(4-ethoxyphenyl)-1,3,4-oxadiazol-2-yl]phenol* (**5c**). The crude solid was recrystallized from ethyl acetate-methanol (1:1) to give a white amorphous solid. Yield 0.371 g (76%), m.p. 176–178 °C, IR (KBr, ν_max_/cm^−1^), 3628 (OH_phenol_), 3009 (CH_aromatic_), 2957 (CH_aliphatic_), 1611 (C=N), 1543, 1495 (C=C), 1221 (C-O), 1111 (O-CH_2_), ^1^H-NMR (CDCl_3_ ,400 MHz, ppm): 1.46 (t, 3H, *J* = 7.32 Hz, OCH_2_CH_3_), 1.51 (s, 18H, H_14_, 2 × *t*-Bu), 4.11 (q, 2H, *J* = 8 Hz, OCH_2_), 5.64 (s, 1H, OH), 7.1 (d, 2H, *J* = 8.8 Hz, H_9_ & H_11_), 7.92 (s, 1H, H_3_), 8.05 (d, 2H, *J* = 8.04 Hz, H_8_ & H_12_), ^13^C-NMR (CDCl_3_, 100 MHz, ppm): 14.71 (OCH_2_CH_3_), 30.17 (6C, C_14_, 2 × C(CH_3_)_3_), 34.48 (2C, C_13_, 2 × C(CH_3_)_3_), 63.73 (OCH_2_), 114.91 (2C, C_8_ & C_12_), 115.50 (C_4_), 116.60 (C_7_), 124.18 (C_3_), 128.57 (2C, C_9_ & C_11_), 136.69 (C_2_), 156.93 (C_1_), 161.52 (C_10_), 164.00 & 164.90 (C_6_ & C_5_). HREIMs, *m/z* = 394.2249 [M^•+^] (calc. for C_24_H_30_O_3_N_2_, 394.2256). 

*4-(5-(4-Bromophenyl)-1,3,4-oxadiazol-2-yl)-2,6-di-tert-butylphenol* (**5d**). The crude product was recrystallized from ethyl acetate-methanol (1-1) to give white crystals. Yield 0.447 g (84%), m.p. 168–170 °C, IR (KBr, ν_max_/cm^−1^): 3525 (OH_phenol_), 3005 (CH_aromatic_), 2953 (CH_aliphatic_), 1602 (C=N), 1543, 1495 (C=C), 1244 (C-O), ^1^H-NMR (CDCl_3_, 400 MHz, ppm): 1.51 (s, 18H, H_14_, 2 × *t*-Bu), 5.66 (s, 1H, OH), 7.67 (d, 2H, *J* = 8.42 Hz, H_9_ & H_11_), 7.92 (s, 2H, H_3_), 7.9 (d, 2H, *J* = 8.54 Hz, H_8_ & H_12_), ^13^C-NMR (CDCl_3_, 100 MHz, ppm): 30.23 (6C, C_14_, 2 × C(CH_3_)_3_), 34.56 (2C, C_13_, 2 × C(CH_3_)_3_), 115.12 (C_4_), 123.22 (C_7_), 124.43 (C_3_), 126.1 (2C, C_8_ & C_12_), 128.31 (2C, C_9_ & C_11_), 132.41 (C_10_), 136.87 (C_2_), 157.33 (C1), 163.37 & 165.66 (C_5_ & C_6_) ppm. HREIMs *m/z* = 428.1093 [M^•+^] (calc. for C_22_H_25_O_2_N_2_Br, 428.1099).

*4-(5-(4-Chlorophenyl)-1,3,4-oxadiazol-2-yl)-2,6-di-tert-butylphenol* (**5e**). The crude material was recrystallized from ethyl acetate-methanol (1:1) to obtain a white solid. Yield 0.396 g (83%), m.p. 162–164 °C, IR (KBr, ν_max_/cm^−1^): 3583 (OH_phenol_), 3004 (CH_aromatic_), 2959 (CH_aliphatic_), 1607 (C=N), 1571, 1540 (C=C), 1239 (C-O). ^1^H-NMR (CDCl_3_-400 MHz, ppm): 1.51 (s, 18H, H_14_, 2 × *t*-Bu), 5.67 (s, 1H, OH), 7.51 (d, 2H, *J* = 8.52 Hz, H_9_ & H_11_), 7.93 (s, 2H, H_3_), 8.07 (d, 2H, *J* = 8.52 Hz, H_8_ & H_12_), ^13^C-NMR (CDCl_3_,100 MHz, ppm): 30.23 (6C, C_14_, 2 × C(CH_3_)_3_), 34.56 (2C, C_13_, 2 × C(CH_3_)_3_), 115 (C_4_), 122.78 (C_7_), 124.27 (C_3_), 129.46 (2C, C_9_ & C_11_) 128.18 (2C, C_8_ & C_12_), 136.87 (C_2_), 137.71 (C_10_), 157.32 (C_1_), 163.29 & 165.64 (C_5_ & C_6_). HREIMs *m/z* = 384.1597 [M^•+^] (calc. for C_22_H_25_O_2_N_2_Cl, 384.1605).

*2,6-Di-tert-butyl-4-(5-(4-hydroxyphenyl)-1,3,4-oxadiazol-2-yl)phenol* (**5f**). The crude mixture was purified by column chromatography using (6:1) hexane ethyl acetate as eluent to give a white amorphous solid. Yield 0.318 g (70%), m.p. 144–146 °C, IR (KBr, ν_max_/cm^−1^): 3617 (OH_phenol_), 2958 (CH_aliphatic_), 1609 (C=N), 1546, 1506 (C=C), 1250 (C-O), ^1^H-NMR (CDCl_3_, 400 MHz, ppm): 1.51 (s, 18H, H_14_, 2 × *t*-Bu), 5.65 (s, 1H, OH), 6.70 (bs, 1H, OH), 7.01 (d, 2H, *J* = 8.76 Hz, H_9_ & H_11_), 7.92 (s, 1H, H_3_), 8.01 (d, 2H, *J* = 8.8 Hz, H_8_ & H_12_), ^13^C-NMR (CDCl_3_, 100 MHz, ppm): 31.06 (6C, C_14_, 2 × C(CH_3_)_3_), 34.56 (2C, C_13_, 2 × C(CH_3_)_3_), 115.27 (C_4_), 115.58 (C_7_), 116.40 (2C, C_8_ & C_12_), 124.37 (C_3_), 128.97 (2C, C_9_ & C_11_), 136.85 (C_2_), 157.24 (C_1_) 159.83 (C_10_), 164.33 & 165.05 (C_5_ & C_6_). HREIMs *m/z* = 366.1938 [M^•+^] (calc. for C_22_H_26_O_3_N_2_, 366.1943).

*2,6-Di-tert-butyl-4-(5-(3,4-dichlorophenyl)-1,3,4-oxadiazol-2-yl)phenol* (**5g**). The crude product was recrystallized from benzene to give a white solid. Yield 0.416 g (80%), m.p. 222–224 °C, IR (KBr, ν_max_/cm^−1^): 3580 (OH_phenol_), 3003(CH_aromatic_), 2952 (CH_aliphatic_), 1606 (C=N), 1546, 1462 (C=C), 1239 (C-O). ^1^H-NMR (CDCl_3_, 400 MHz, ppm): 1.44 (s, 18H, H_14_, 2 × *t*-Bu), 5.67 (s, 1H, OH), 7.59 (d, 2H, *J* = 8.52 Hz, H_11_), 7.91–7.95 (m, 3H, H_12_ & H_3'_), 8.19 (d, *J* = 2.2 Hz, 1H, H_8_), ^13^C-NMR (CDCl_3_, 100 MHz, ppm): 30.23 (6C, C_14_, 2 × C(CH_3_)_3_), 34.57 (2C, C_13_, 2 × C(CH_3_)_3_), 114.89 (C_4_), 124.11 (C_7_), 124.51 (C_3_), 125.95 (C_12_), 128.53 (C_11_), 131.28 (C_8_), 133.68 (C_9_), 135.89 (C_10_), 136.94 (C_2_), 157.50 (C_1_), 162.3 & 165.94 (C_5_ & C_6_). HREIMs *m/z* = 418.1219[M^•+^] (calc. for C_22_H_24_O_2_N_2_Cl_2_, 418.1215).

*2,6-Di-tert-butyl-4-(5-(3,5-dichlorophenyl)-1,3,4-oxadiazol-2-yl)phenol* (**5h**). The crude product was purified by recrystallized from acetonitrile to afford a white amorphous solid. Yield 0.386 g (74%), m.p. 195–197 °C, IR (KBr, ν_max_/cm^−1^): 3600 (OH_phenol_), 3007 (CH_aromatic_), 2961 (CH_aliphatic_), 1606 (C=N), 1574, 1550 (C=C), 1240 (C-O), ^1^H-NMR (CDCl_3_, 400 MHz, ppm): 1.52 (s, 18H, H_14_, 2 × *t*-Bu), 5.69 (s, 1H, OH), 7.51 (t, 1H, *J* = 1.24 Hz, H_10_), 7.93 (s, 2H, H_3_), 8.01 (t, 2H, *J* = 1.72 Hz, H_8_ & H_12_), ^13^C-NMR (CDCl_3_, 100 MHz, ppm): 30.39 (6C, C_14_, 2 × C(CH_3_)_3_), 34.50 (2C, C_13_, 2 × C(CH_3_)_3_), 114.73 (C_4_), 124.48 (C_3_), 125.02 (2C, C_8_ & C_12_), 126.87 (C_7_), 131.24 (C_10_), 135.92 (C_9_ & C_11_), 136.93 (C_2_), 157.50 (C_1_), 161.877 (C_5_), 166.05 (C6). HREIMs *m/z* = 418.1210 [M^•+^] (calc. for C_22_H_24_O_2_N_2_Cl_2_, 418.1215).

*2,6-Di-tert-butyl-4-(5-(2,4-dimethylphenyl)-1,3,4-oxadiazol-2-yl)phenol* (**5i**). The crude product was purified by recrystallized from toluene to give white crystalline needles. Yield 0.28 g (60%), m.p. 170–172 °C. IR (KBr, ν_max_/cm^−1^): 3587 (OH_phenol_), 2957 (CH_aliphatic_), 1614 (C=N), 1550, 1536 (C=C), 1238 (C-O), ^1^H-NMR (CDCl_3_, 400 MHz, ppm): 1.50 (s, 18H, H_14_, 2 × *t*-Bu), 2.39 (s, 3H, H_15_), 2.70 (s, 3H, H_16_), 5.64 (s, 1H, OH), 7.17–7.14 (m, 2H, H_9_ & H_11_), 7.95–7.90 (m, 3H, H_12_, H_3'_), ^13^C-NMR (CDCl_3_, 100 MHz, ppm): 21.50 (C_16_, *o*-CH_3_), 22.04 (C_15_, *p*-CH_3_), 30.22 (6C, C_14_, 2 × C(CH_3_)_3_), 34.55 (2C, C_13_, 2 × C(CH_3_)_3_), 115.40 (C_4_), 12.60 (C_7_), 124.38 (C_3_), 126.98 (C_11_), 128.96 (C_12_), 132.59 (C_9_), 136.79 (C_2_), 138.19 (C_8_), 141.43 (C_10_), 157.11 (C_1_), 164.49 & 164.81 (C_5_ & C_6_). HREIMs *m/z* =378.2301 [M^•+^] (calc. for C_24_H_30_O_2_N_2_, 378.2307).

*2,6-Di-tert-butyl-4-[5-(2-methylphenyl)-1,3,4-oxadiazol-2-yl] phenol* (**5j**). The product was recrystallized from ethyl acetate to afford white crystals, 0.308 g (68.5%), m.p. 132–134 °C, IR (KBr, ν_max_/cm^−1^): 3588 (OH_phenol_), 3008 (CH_aromatic_), 2963 (CH_aliphatic_), 1607 (C=N), 1592, 1537 (C=C), 1238 (C-O), ^1^H-NMR (CDCl_3_,400 MHz, ppm): 1.52 (s, 18H, H_14_, 2 × *t*-Bu), 5.66 (s, 1H, OH), 7.44–7.34 (m, 3H, H_9_, H_10_, H_11_), 7.96 (s, 2H, H_3_, H_3'_), 8.02 (d, 1H, *J* = 7.32 Hz, H_12_), ^13^C-NMR (CDCl_3_, 100 MHz, ppm): 2 2.11 (*o*-CH_3_), 30.22 (6C, C_14_, 2 × C(CH_3_)_3_), 34.52 (2C, C_13_, 2 × C(CH_3_)_3_), 115.53 (C_4_), 123.40 (C_7_), 124.37 (C_3_), 126.21 (C_11_), 128.96 (C_12_), 131.03 (C_10_), 131.80 (C_9_), 136.80 (C_2_), 138.35 (C_8_), 157.14 (C_1_), 164.32 & 165.04 (C_5_ & C_6_), HREIMs *m/z* = 364.2144 [M^•+^] (calc. for C_23_H_28_O_2_N_2_, 364.2151).

*2,6-Di-tert-butyl-4-(5-(2-chlorophenyl)-1,3,4-oxadiazol-2-yl)phenol* (**5k**). The crude product was recrystallized from ethyl acetate to obtain white crystals. Yield 0.386 g (81%), m.p. 113–115 °C, IR (KBr, ν_max_/cm^−1^): 3584 (OH_phenol_), 3004 (CH_aromatic_), 2959 (CH_aliphatic_), 1607 (C=N), 1570, 1539 (C=C), 1239 (C-O), ^1^H-NMR (CDCl_3_, 400 MHz, ppm): 1.48(s, 18H, H_14_, 2 × *t*-Bu), 5.66 (s, 1H, OH), 7.38–7.46 (m, 2H, H_10_, H_11_), 7.53 (d, 1H, *J* = 8.04 Hz, H_12_), 7.95 (s, 2H, H_3_, H_3'_), 8.05 (d, 1H, *J* = 6.32 Hz, H_9_), ^13^C-NMR (CDCl_3_, 100 MHz, ppm): 30.27 (6C, C_14_, 2 × C(CH_3_)_3_), 34.61 (2C, C_13_, 2 × C(CH_3_)_3_), 115.18 (C_4_), 123.65 (C_7_), 124.61 (C_3_), 127.21 (C_11_), 131.33 (C_12_), 132.32 (C_9_), 133.14 (C_8_), 136.79 (C_9_), 136.94 (C_2_), 157.28 (C_1_), 162.55 & 166.04 (C_5_ & C_6_). HREIMs *m/z* = 384.1600 [M^•+^] (calc. for C_22_H_25_O_2_N_2_Cl, 384.1605).

## 5. Conclusions

A series of new 1,3,4-oxadiazole compound incorporating hindered phenol moities were successfully synthesized and characterized. All of the new compounds were screened for antioxidant activity using the FRAP and DPPH assays. The substituents on ring C demonstrated a significant role in improving or negating the antioxidant activity of the compounds. The analogues incorporating electron releasing substituents exhibited high antioxidant activity, whereas those with electron withdrawing substituent demonstrated reduced antioxidant activity.

## Supplementary Materials

Supplementary materials can be accessed at: http://www.mdpi.com/1420-3049/19/3/3436/s1.
