# Supplementary material for: Synthesis of New 2,5-Di-substituted 1,3,4-Oxadiazoles Bearing 2,6-Di-tert-butylphenol Moieties and Evaluation of Their Antioxidant Activity
Source: Molecules. 2014 Mar 20;19(3):3436–49. doi: 10.3390/molecules19033436 (PMC6271237; doi:10.3390/molecules19033436)

Supplementary Materials

Figure S1. ^{1}H-NMR (400 MHz, $CDCl_3$) of **5d**.

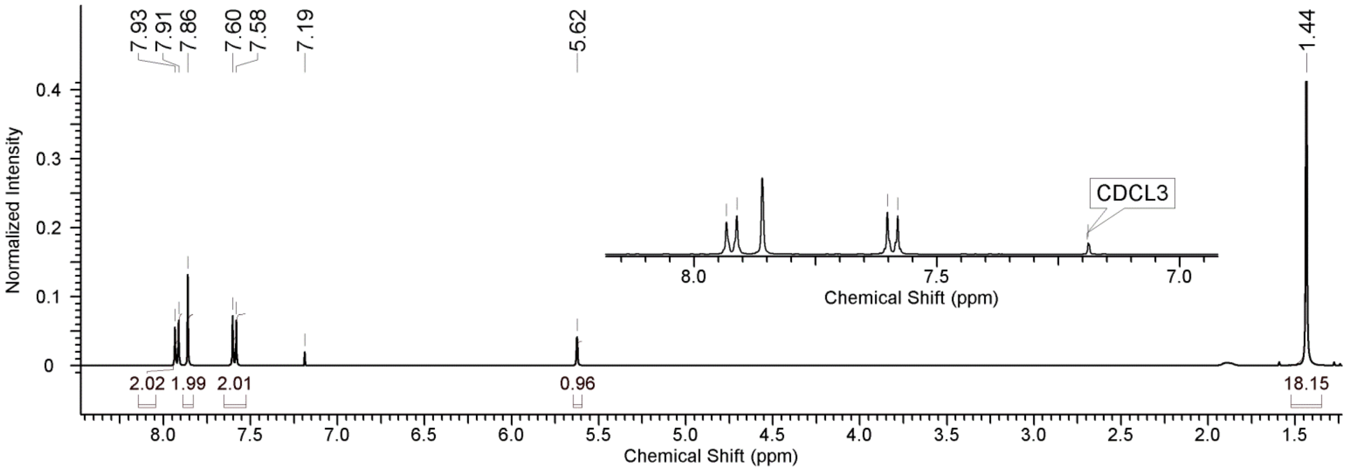

Figure S2. ^{13}C-NMR (100 MHz, $CDCl_3$) of **5d**.

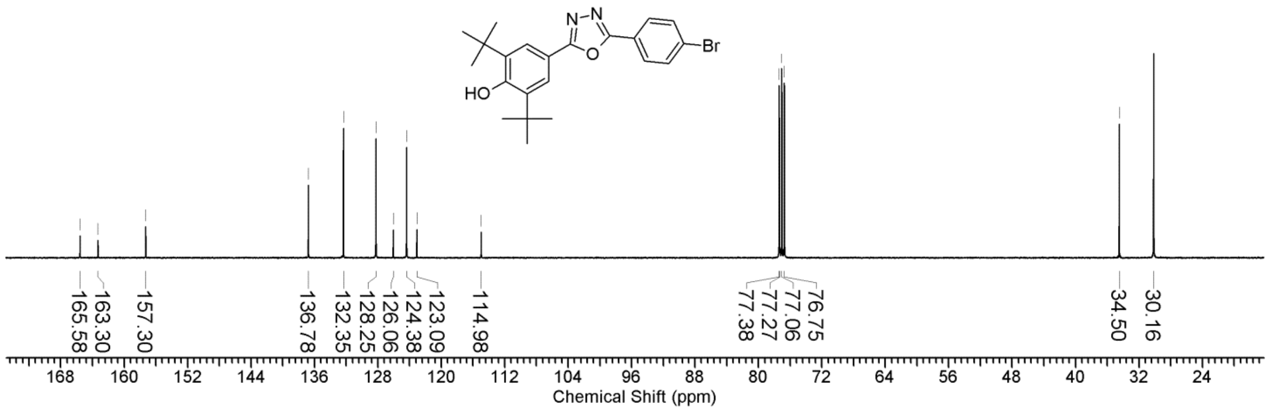

Figure S3. EIMS spectrum of **5a**.

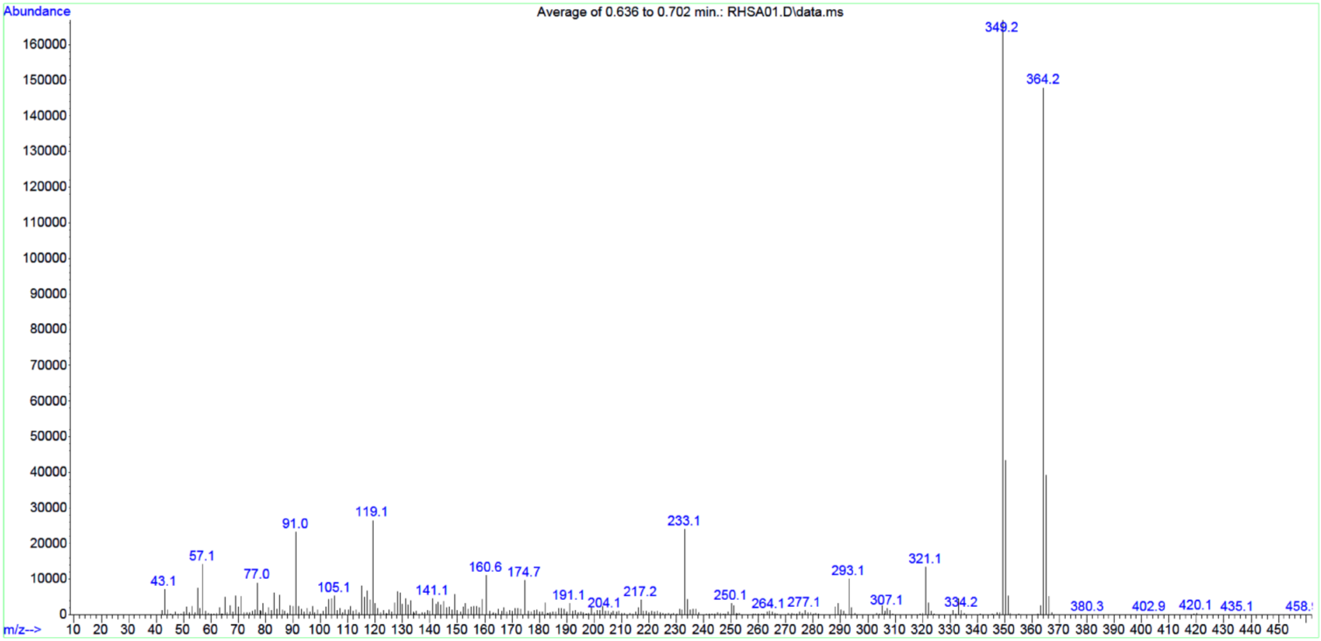

Figure S4. EIMS spectrum of **5b**.

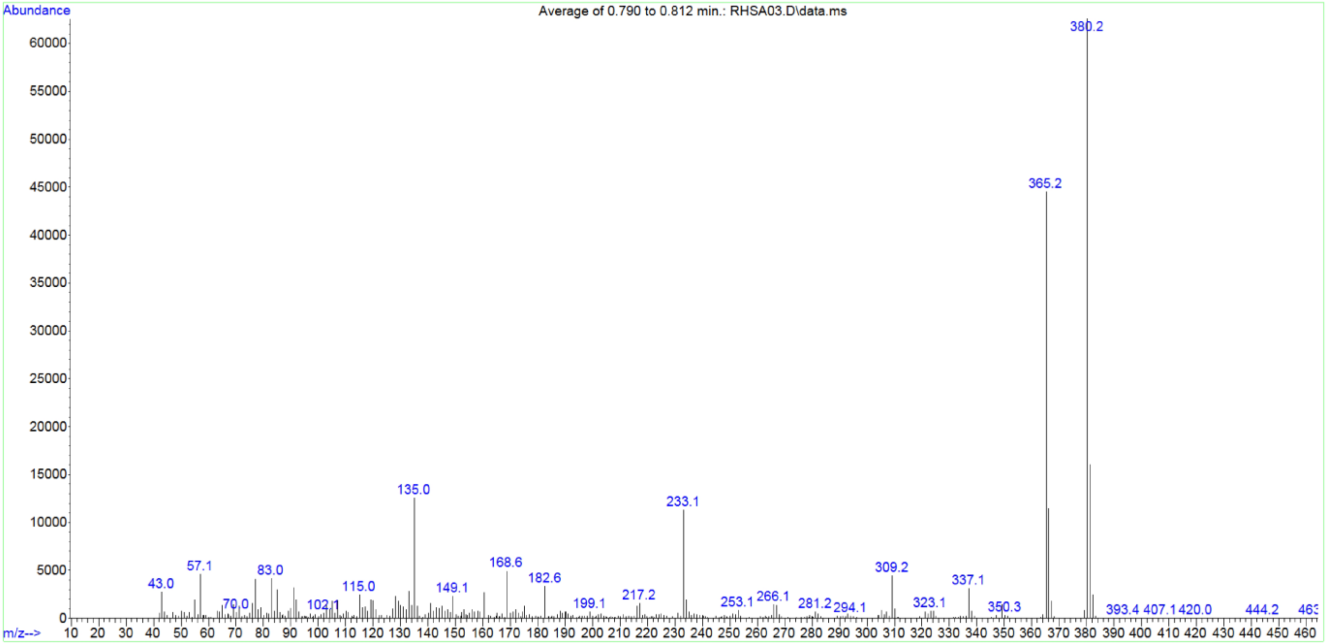

Figure S5. EIMS spectrum of **5c**.

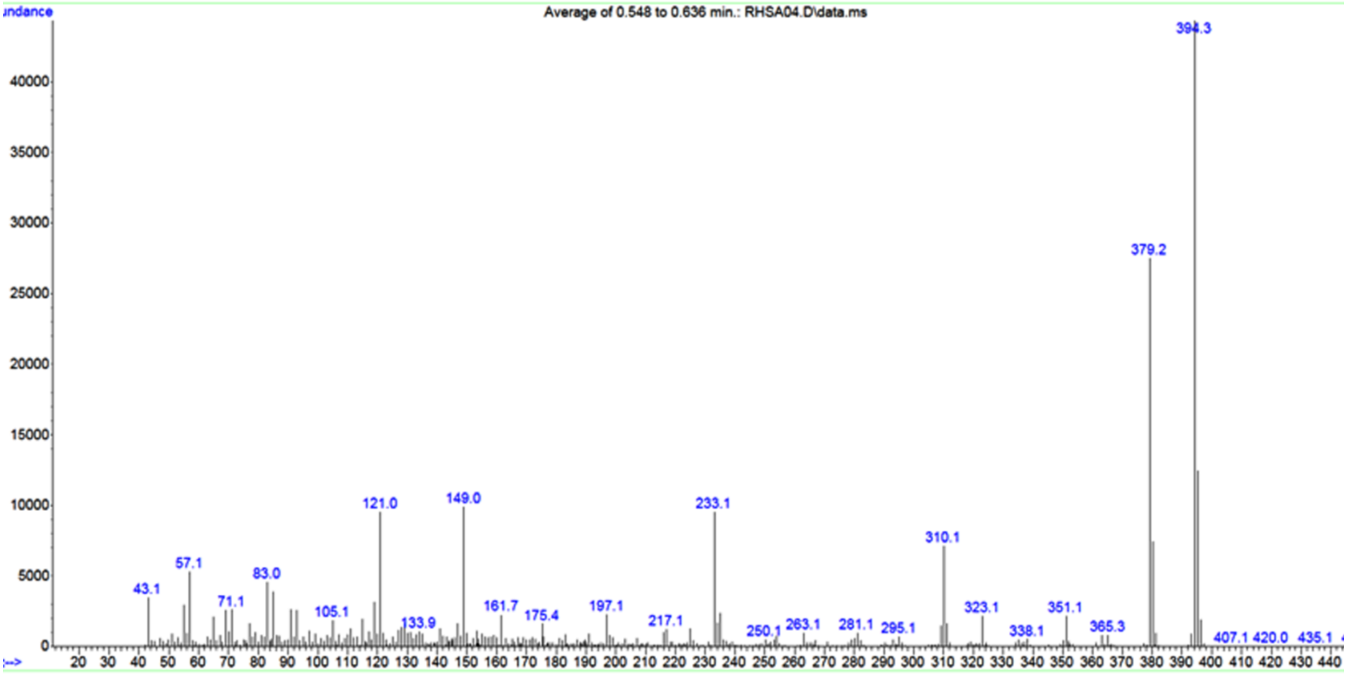

Figure S6. EIMS spectrum of **5f**.

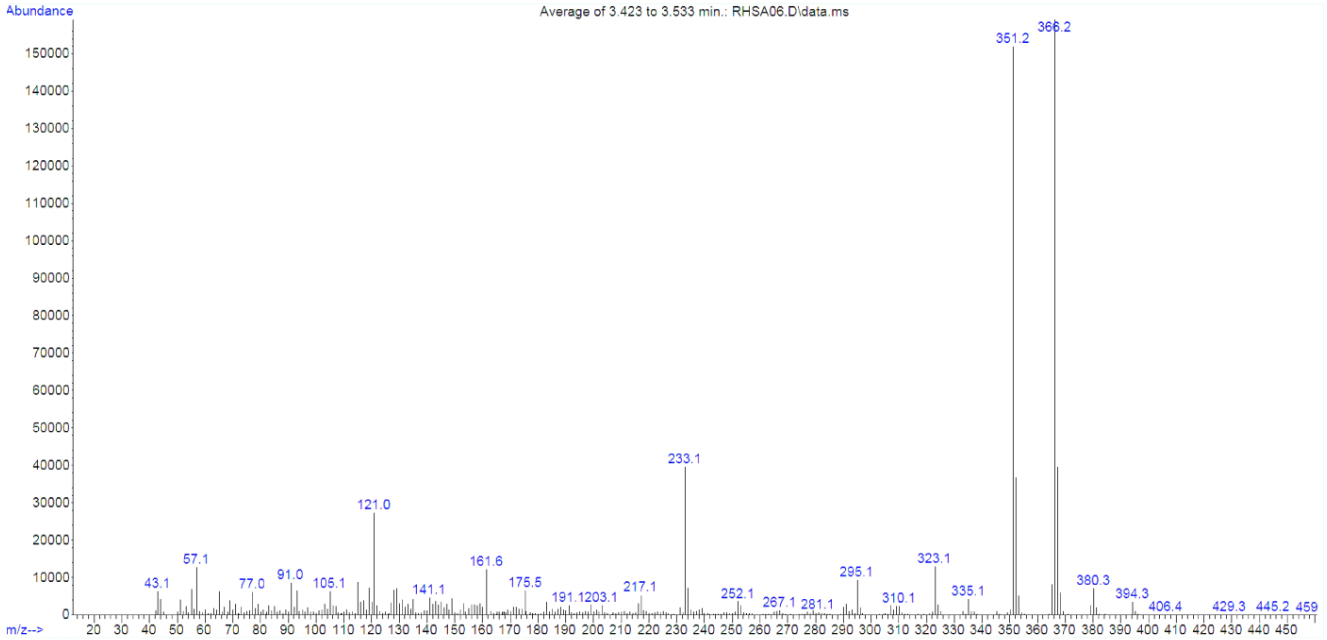

Figure S7. EIMS spectrum of **5h**.

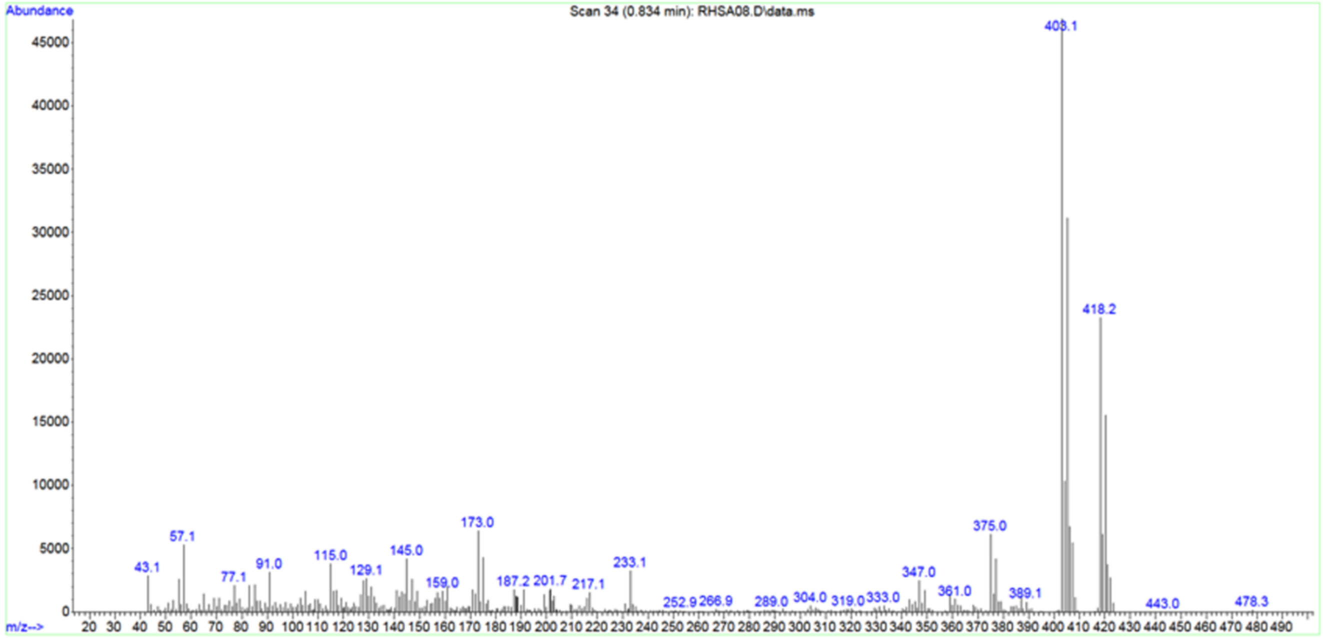

Figure S8. EIMS spectrum of **5i**.

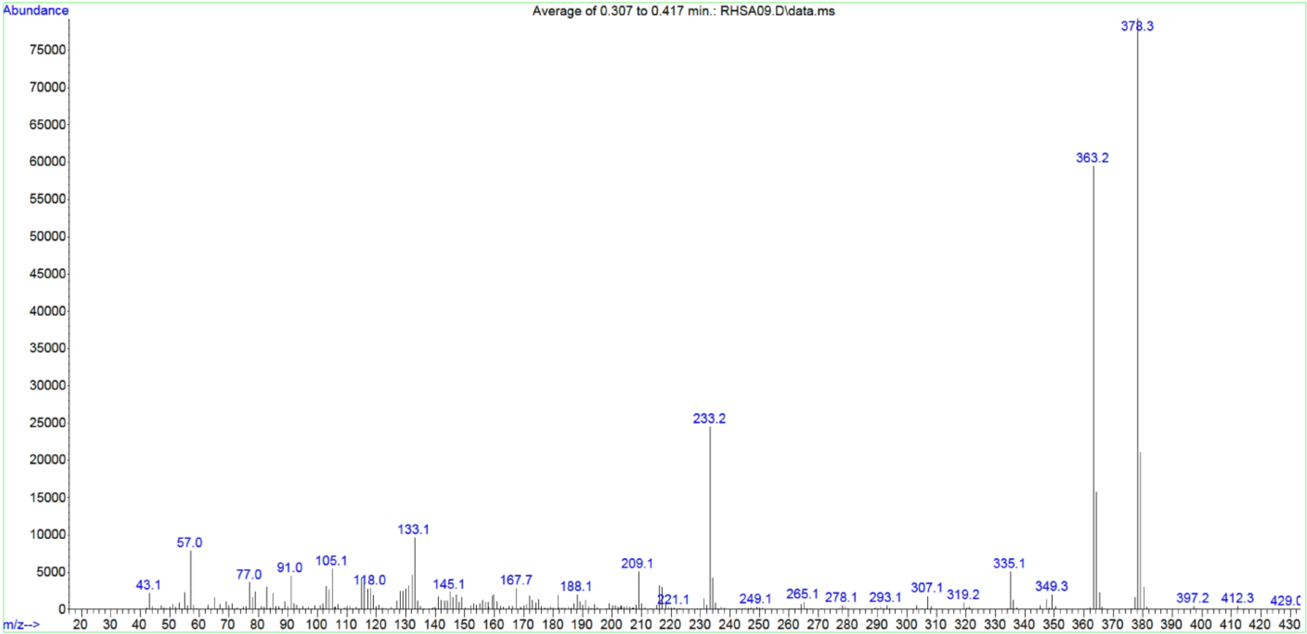

Supplement: Supplementary file 1 [file molecules-19-03436-s001.pdf]
